# Ferroptosis—A Shared Mechanism for Parkinson’s Disease and Type 2 Diabetes

**DOI:** 10.3390/ijms25168838

**Published:** 2024-08-14

**Authors:** Carmen Duță, Corina Muscurel, Carmen Beatrice Dogaru, Irina Stoian

**Affiliations:** Department of Biochemistry, Carol Davila University of Medicine and Pharmacy, 050474 Bucharest, Romania; corina.muscurel@umfcd.ro (C.M.); carmenbeatrice.dogaru@umfcd.ro (C.B.D.); irina.stoian@umfcd.ro (I.S.)

**Keywords:** Parkinson’s disease, type 2 diabetes, oxidative stress, iron metabolism, ferroptosis

## Abstract

Type 2 diabetes (T2D) and Parkinson’s disease (PD) are the two most frequent age-related chronic diseases. There are many similarities between the two diseases: both are chronic diseases; both are the result of a decrease in a specific substance—insulin in T2D and dopamine in PD; and both are caused by the destruction of specific cells—beta pancreatic cells in T2D and dopaminergic neurons in PD. Recent epidemiological and experimental studies have found that there are common underlying mechanisms in the pathophysiology of T2D and PD: chronic inflammation, mitochondrial dysfunction, impaired protein handling and ferroptosis. Epidemiological research has indicated that there is a higher risk of PD in individuals with T2D. Moreover, clinical studies have observed that the symptoms of Parkinson’s disease worsen significantly after the onset of T2D. This article provides an up-to-date review on the intricate interplay between oxidative stress, reactive oxygen species (ROS) and ferroptosis in PD and T2D. By understanding the shared molecular pathways and how they can be modulated, we can develop more effective therapies, or we can repurpose existing drugs to improve patient outcomes in both disorders.

## 1. Introduction

The occurrence of PD is approximately 0.15% among the general population, but in the population aged 65 years and over, the incidence rate is 1.5% [1] and above 85 years increases to 4–5% [2]. Additionally, it is predicted that the number of individuals impacted by PD will double in the next three decades due to its growing prevalence in an aging population [3].

At present, the term “Parkinson’s disease” implies three stages [4]:

(1) The preclinical phase, which is the early stage when the neurodegenerative process started, but no symptoms are evident yet;
(2) The prodromal phase, which is characterized by the progression of degeneration in various regions of the central and peripheral nervous system, leading to clinically evident symptoms;
(3) The clinical phase, which is the stage when the typical motor symptoms are prominent enough to confirm diagnosis of PD.

The typical age when symptoms of PD begin was previously thought to be in the late fifties. However, due to a rise in the average age of people, the onset of symptoms is now commonly observed in the sixties [4]. It appears that the preclinical and prodromal phases have an average duration of 15 years, before the initial noticeable symptoms of PD, such as tremors, appear [5].

The prodromal phase is important for PD research because intervening during this phase could potentially alter the disease’s course before a substantial loss of dopaminergic neurons in the brain occurs. Many of the prodromal markers are found frequently in PD patients and have high sensitivity, but they have low specificity, as they also often occur in the general population [6]. The Movement Disorder Society (MDS) gathered these markers in 2015 [7] in “Research Criteria on Prodromal Parkinson’s Disease”, which was updated in 2019 [8], and a free online calculator is also available for research groups (http://www.movementdisorders.org/pdcalculator, accessed on 14 February 2024).

The primary clinical indicators of the disease are bradykinesia, muscle rigidity, resting asymmetric tremors in the upper limbs, gait difficulties, inability to initiate voluntary movements and postural instability [4,9]. These symptoms occur because of the gradual degeneration of the substantia nigra pars compacta (SNc). PD symptoms become noticeable when approximately 50% of the neurons in the substantia nigra (SN) have died [10]. This loss of half of the SN results in a reduction in dopamine (DA) concentration, which is 20% of the normal level at the onset of symptoms and the SN becomes pale [3]. Degeneration affects neurons throughout the basal ganglia, olfactory bulbs, sympathetic ganglia and dopaminergic neurons in the gut [11]. Nonetheless, approximately 30–40% of PD cases do not exhibit tremors, and this may lead to as much as half of all cases remaining undiagnosed [12].

Non-motor symptoms include dementia, depression, social phobia, anxiety, loss of smell, fear, sleep disorders, mood disorders, cognitive impairment, gastrointestinal symptoms, pain and autonomic symptoms [10,11,12].

Various mechanisms can cause the death of nervous cells, which can be triggered by genetic (10% of the cases) and environmental factors. Some studies revealed that depression and stress can play an essential role in PD incidence [13].

Although there are various treatments available for PD, they only provide relief from the symptoms, and no cure for the disease currently exists.

Diabetes mellitus is, according to the WHO, “a chronic, metabolic disease characterized by elevated levels of blood glucose, which leads over time to serious damage to the heart, blood vessels, eyes, kidney, and nerves” and is affecting over 400 million people, 85–90% having T2D [14]. T2D is characterized by the inability of insulin-sensitive tissues to respond to insulin (insulin resistance) and a gradual decrease in insulin production due to a reduction in beta-cell function and number in the pancreas. Numerous diabetic complications through various mechanisms are caused by insulin resistance and hyperglycemia [15].

Epidemiological studies suggest that individuals with T2D are at increased risk of developing PD [16]. Furthermore, clinical research has found that PD’s symptoms tend to worsen considerably following the onset of T2D [14]. Several pathological and molecular connections are shared by both PD and T2D: insulin resistance, oxidative stress, mitochondrial dysfunctions, neuroinflammation and protein misfolding [2]. Ultimately, these mechanisms trigger neuronal and pancreatic cell dysfunction and death.

In this review, we will discuss iron accumulation in the brain and pancreas in PD and T2D cases, iron’s implication in generating ROS, their implication in lipid peroxidation and DNA damage and the major pathways regulating ferroptosis. We will not consider other forms of regulated cell death, which are also common for the two diseases.

## 2. Ferroptosis

Before introducing the term of ferroptosis, erastin was identified in 2003 as a synthetic lethal small molecule in human foreskin fibroblasts (BJeLR) expressing mutant Ras oncogene but not in normal cells [17]. Later, in 2008, RSL3 and RSL5 killed BJeLR cells in a non-apoptotic manner [18]. RSL-induced death cannot be reversed by inhibition of apoptosis, necrosis and autophagy, but, in contrast, antioxidants (e.g., vitamin E) and iron chelators (e.g., deferoxamine mesylate) block cell death [19].

In 2012, Brent R. Stockwell introduced a distinct type of regulated cell death characterized by the excessive accumulation of lipid-based ROS, which is dependent on iron. This unique form of cell death was named “ferroptosis” [19]. Ferroptosis exhibits clear morphological and biochemical differences compared to other forms of regulated cell death. Unlike apoptosis, ferroptosis does not display chromatin condensation or nuclear reduction, and it lacks the cellular and organellar swelling typical of necroptosis. Additionally, it does not exhibit the common features associated with autophagy. The primary morphological distinction is mitochondrial shrinkage [20,21], disappearance of mitochondrial cristae and mitochondrial outer membrane rupture [22,23]. Cytoplasmic swelling (oncosis) and plasma membrane rupture resemble necrosis [19]. The main morphological changes in ferroptosis, apoptosis, necroptosis and autophagy are presented in Table 1 (adapted from [24]).

The involvement of mitochondria in ferroptosis remains a subject of debate, despite the marked alterations in its structure. In contrast to an earlier study suggesting that mitochondria ROS production is not essential for ferroptosis [19], recent findings highlight the necessity of mitochondrion-mediated ROS production, DNA stress and metabolic reprogramming for lipid peroxidation and the initiation of ferroptosis.

Ferroptotic cell death is closely associated with an iron-dependent mechanism that triggers the generation of highly reactive free radicals, resulting in extensive peroxidation of membrane phospholipids (PLs) rich in polyunsaturated fatty acids (PUFAs).

There are three main factors that determine cell death by ferroptosis: (1) an increase in free intracellular iron, (2) depletion of the antioxidant system that removes and controls oxidative stress, mainly glutathione, glutathione peroxidase enzyme (GPx4) and system xc^−^, and (3) the peroxidation of membrane phospholipids (PLs) rich in PUFAs. The main PLs affected are those containing arachidonic or adrenic acids derived from phosphatidyl ethanolamine (PE) molecules [23,25,26].

In 2018, the Nomenclature Committee on Cell Death (NCCD) defined ferroptosis as “a form of RCD (regulated cell death) initiated by oxidative perturbations of the intracellular microenvironment that is under constitutive control by GPx4 and which can be inhibited by iron chelators and lipophilic antioxidants” [27].

### 2.1. Iron Homeostasis

Iron is a crucial trace element for the functioning of living organisms, as it plays a vital role in various metabolic processes including oxygen transport, energy metabolism, nucleotide synthesis and electron transport [28]. However, excessive levels of iron can be harmful, so it is necessary to maintain its concentration within an optimal range. In humans, iron homeostasis is achieved by carefully balancing the uptake of iron with its intracellular utilization and storage [28].

The regulation of iron metabolism has been the subject of comprehensive reviews [28,29,30,31,32,33], and a summary will be provided here. In mammalian organisms, approximately 20–25 mg iron per day is recycled from aging red blood cells. A smaller fraction, around 5–10% of that amount, is absorbed from the intestine. Unlike some other organisms, mammals lack the ability to regulate the excretion of excess iron. Under normal conditions, the loss of iron through processes such as shedding of intestinal epithelial cells, cell death and biliary excretion balances out the intestinal uptake. However, when the uptake of iron exceeds its loss, the surplus iron is stored intracellularly.

**Figure 1:**
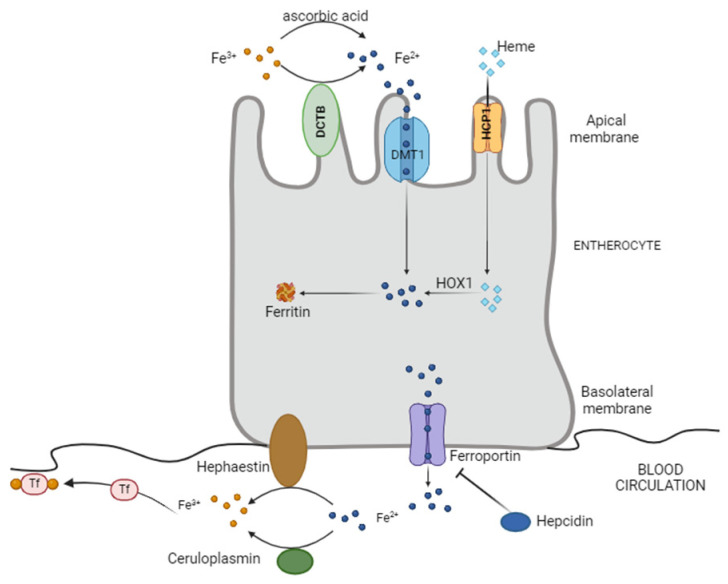
Intestinal absorption of iron, storage and export. Fe^3+^ is reduced to Fe^2+^, in the duodenum, by the ferrireductase duodenal cytochrome b (DCTB) and enters the cell with the help of divalent metal-ion transporter 1 (DMT1, also known as SCL11A2, NRAMP2 and DCT1). Heme iron is transported into the enterocytes by the heme carrier protein 1 (HCP-1), is detached by heme oxygenase (mainly the inducible hemoxigenase 1-HOX1) and then enters the same storage and transport pathways as inorganic iron. Iron is either stored within the cell as ferritin (FT) or is exported across the basolateral membrane of the enterocyte. Ferroportin (FPN, also known as SLC40A1, IREG1 and MTP1) is the only protein capable of releasing Fe^2+^ into the blood capillary. Fe^2+^ is then oxidized into Fe^3+^ by ceruloplasmin in most of the cells or hephaestin (HEPH) in enterocytes and binds to transferrin (Tf). Created with BioRender.com. (accessed on 7 June 2024).

Mammals only obtain iron from food: inorganic iron, non-heme iron, is present in a wide variety of foods but is inefficiently absorbed, and heme iron, mainly as hemoglobin and myoglobin, is more efficiently absorbed. Figure 1 presents the absorption, storage and export of iron in the enterocyte.

**Figure 2:**
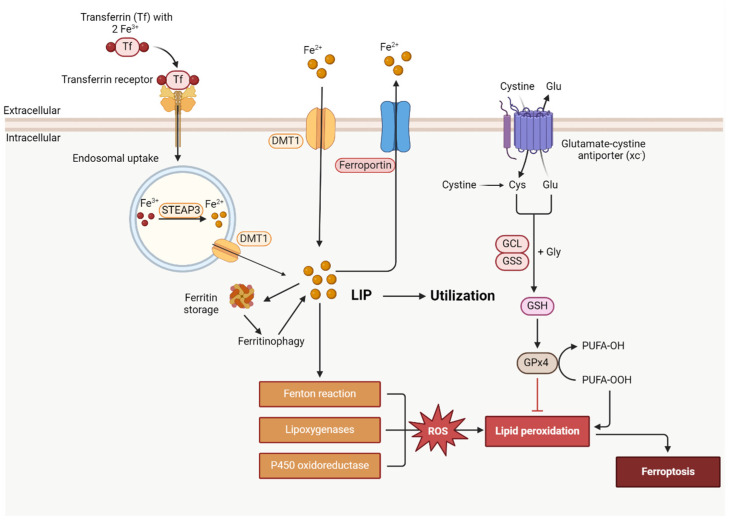
Metabolic pathways that contribute to ferroptosis. DMT1—divalent metal-ion transporter 1; Tf—transferrin; STEAP3—six-transmembrane epithelial antigen of prostate 3; GCL—glutamate cysteine ligase; GSS—glutathione synthase; GSH—glutathione; GPx4—glutathione peroxidase 4; PUFA—polyunsaturated fatty acid; ROS—reactive oxygen species; LIP—labile iron pool. Adapted from [34]. Created with BioRender.com (accessed on 7 June 2024).

Transferrin carries iron to all the organs and tissues. Transferrin receptors (TfRs, in most cells TfR1) mediate iron uptake in most cells. After endocytosis, the endosome is acidified and Fe^3+^ is released from transferrin and is then reduced to Fe^2+^ by STEAP3 (six-transmembrane epithelial antigen of prostate 3) and released through DMT1 into the labile iron pool (LIP) (Figure 2).

LIP is utilized for either direct integration into iron-utilizing proteins or for transferring iron to mitochondria using mitoferrin (Mfrn), where the element is incorporated into heme and Fe/S cluster prosthetic units. The portion of the LIP not used can either be transported out through ferroportin, in collaboration with ferroxidases that load iron onto transferrin, or be stored safely in ferritin capsules. Ferritin can be released into the extracellular environment through mechanisms that are not yet understood and can interact with specific receptors on the cell’s surface. Some cells also have a variant of mitochondrial ferritin, which shields the organelle from iron-related harm. The LIP’s size is determined by the speed of iron absorption, utilization, storage and export. These processes need to be harmoniously controlled to prevent detrimental iron deficiency and avoid excessive iron accumulation. LIP regulation will be discussed later in the article.

The factors that are recognized to influence the iron homeostatic system include the iron requirements for erythropoiesis, low oxygen levels (hypoxia), insufficient iron levels, excessive iron levels and inflammatory responses. It has been revealed that a circulating peptide hormone called hepcidin is largely responsible for governing a significant portion of this regulatory process [35,36,37]. Hepcidin interrupts cellular iron export by binding directly to ferroportin in at least two sites: the intestinal epithelium and tissue macrophages. The complex is then phosphorylated by Jak2, and afterwards it is internalized, ubiquitinated and degraded within lysosomes [38]. The expression of hepcidin changes in reaction to various triggers that impact the balance of iron within the body. It rises when there is a surplus of iron in the blood, due to iron overload, inflammation or elevated serum iron levels. Conversely, it decreases when there is a higher demand for red blood cell production, a lack of oxygen (hypoxia) or a shortage of iron (iron deficiency) [37,39,40].

### 2.2. The Labile Iron Pool (LIP)

The term “labile iron pool” was proposed in 1946 [41] and reintroduced in 1977 as a “transient iron pool” [42], between extracellular iron and cellular iron associated with proteins. It consists of both ionic forms of iron (Fe^2+^ and Fe^3+^) weakly associated with a variety of low-molecular weight ligands: organic anions (phosphates, citrates and carboxylates), polypeptides, phospholipids, carbohydrates, nucleotides and nucleosides [43]. It is also exchangeable, being easily transferred among natural ligands and between cell compartments [44].

LIP represents 3–5% of the total cellular iron and can undergo dynamic shifts due to biochemical stimuli, and the resultant alterations are typically temporary and aimed at maintaining stability within the system [43]. Nonetheless, during severe instances of excessive or insufficient iron levels, these modifications could surpass the cell’s ability to maintain equilibrium, potentially jeopardizing its overall integrity [43].

Iron uptake is the major source of LIP [45], and other sources are proteins containing iron within cells, such as ferritin and hemeproteins.

Ferritin is a heteropolymer capable of storing 4500 Fe^3+^ per molecule, made of 24 subunits of heavy (FtH1) and light (FtL) chains, where hydrated iron oxides are held in cage-like nanocavities [46]. Their expression ratios differ based on the specific cell type and are influenced by factors like inflammation or infection. FtH1 is responsible for the ferroxidase activity, essential for depositing iron within the nanocage structure. On the other hand, FtL aids in iron nucleation and enhances the activity turnover of the ferroxidase site [31]. Ferritin serves a dual function in maintaining LIP balance. Its primary role involves capturing iron, safeguarding cells from iron-related harm. Elevating the heavy subunit of ferritin leads to a reduction in LIP levels [47] and lessens DNA damage caused by H_2_O_2_ [48]. The processes by which iron is liberated from ferritin are not well understood, and it appears that the predominant method of iron release involves the degradation of the protein within lysosomes (ferritinophagy) [49,50]. There is also limited understanding regarding the mechanisms involved in removing iron from the LIP and transferring it to ferritin. Poly(rC)-binding protein 1 (PCBP1), an RNA-binding protein, primarily recognized for its involvement in post-transcriptional control, is essential for facilitating the loading of iron into ferritin in cultured cells and has demonstrated the ability to encourage this process in laboratory settings [51].

Little is known about non-heme iron proteins involved in LIP formation. Among them, iron–sulfur proteins have major importance. They are characterized by one or more non-heme iron ions that are bound to sulfur atoms, creating planar or cubic clusters of Fe-S. These iron–sulfur proteins primarily serve the purpose of facilitating electron transfer and are involved in a diverse range of cellular functions [52]. The functionality of numerous Fe-S cluster proteins, particularly those with 4Fe-4S configurations, can be influenced by oxidative stress. Hydrogen peroxide quickly interacts with the 4Fe-4S clusters found in the cytosolic aconitase of mammalian cells, causing the enzyme to shift into an inactive 3Fe-4S state [53]. Consequently, the iron ion that is released likely enters the LIP.

There seems to exist also small subcellular LIP in the mitochondria, nucleus and lysosomes (likely those degrading iron-containing proteins) of several cells [43,44].

The available data suggest that the LIP levels exhibit variations among various cell types and are not uniformly distributed within the cell. Recent findings by Lipinski et al. [54] highlight that even closely related cell lines could display discrepancies in diverse facets of iron regulation. These dissimilarities within and between cells probably arise from the distinct roles of specific organelles, their requirements for iron ions and the unique functions of various cell types within the entire organism.

#### 2.2.1. LIP Regulation

Observations of LIP concentrations in cultured cells have suggested that these levels are controlled within a relatively narrow range of concentrations [55,56]. It is presumed that this regulation is upheld within cells to ensure they fulfil their metabolic iron requirements while reducing the likelihood of contributing to ROS generation.

#### 2.2.2. IRP-Dependent Control of LIP

Cytosolic levels of LIP are thought to be detected by iron-responsive proteins (IRPs), members of the aconitase family of enzymes [57], which then prompt corrective measures. IRP1 is a bifunctional protein capable of integrating a 4Fe-4S cluster and serves as an iron detector [58]. In contrast, IRP2 lacks an iron–sulfur cluster and aconitase activity and functions solely as an mRNA-binding protein [43].

In situations of low LIP levels, IRP1 loses the 4Fe-4S cluster, and this induces an mRNA-binding activity of the enzyme. IRP1 attaches to a palindromic hairpin structure called iron-responsive elements (IREs) located at the untranslated regions (UTRs) of primarily two groups of mRNAs that encode for iron transport and storage: (i) extracellular iron transporters, such as the transferrin receptor (TfR) and the transmembrane divalent cation transporter 1 (DCT1); and (ii) internal iron storage proteins like ferritin.

At high levels of LIP, IRP1 is unable to bind RNA, taking instead an enzymatic role as a cytoplasmic aconitase [59]. When iron levels are replenished, causing an increase in LIP, IRPs sense this change, binding to iron and becoming inactive. Consequently, the binding of IRPs to IRE diminishes, leading to elevated ferritin translation while hindering the synthesis of iron transporters. At high LIP, IRP2 undergoes ubiquitination and degradation [60].

The interaction between IRPs and IREs serves one of two purposes, depending on the location of the IREs. When IRPs bind to IREs situated in the 5′ untranslated regions of mRNAs encoding ferritin, ferroportin and the heme biosynthetic enzyme aminolevulinate synthase, it obstructs the initiation of translation by disrupting ribosome assembly at the starting codon. In the context of ferritin, this disruption halts the generation of both ferritin subunits, leading to a decrease in iron storage capacity, especially when storing iron would be counterproductive [43]. IREs located in the 3′ untranslated region of TfR1 mRNA fulfil an entirely different role. In this scenario, five consecutive IREs attach to IRP, thereby enhancing the stability of the molecule through inhibition of nuclease degradation. This safeguard allows for increased production of TfR1 when iron availability is limited. A parallel function, likely carried out by a singular 3′ UTR, is presumed to be present in two out of four potential isoforms of DMT1 mRNA [61].

#### 2.2.3. IRP-Independent Control of LIP

Lipinski and colleagues [31] introduced an intriguing cellular model featuring two closely related mouse lymphoma cell lines that exhibit contrasting levels of LIP. In these lines, the determining factor for LIP levels is the transcription and expression of IRP1 protein: higher protein expression necessitates increased iron to counteract its IRE-binding activity. Additionally, the regulation of LIP can also involve the expression of ferritin and/or DMT1, with these mechanisms not being mutually exclusive. In the mentioned model, elevated IRP1 expression coincides with reduced FT expression. Demonstrating this, overexpression of FtH led to decreased LIP levels in MEL cells [47], whereas DMT1 overexpression increased LIP levels in CHO cells [62]. Hence, it appears that the expression of key iron homeostasis proteins like IRP1, ferritin and/or DMT1 establishes cellular LIP levels, while IRP/IRE binding maintains the stability of these levels.

Cytokines are believed to impact the LIP through various mechanisms that vary depending on the specific cell and cytokine involved. For instance, cytokines like TNF-α and interleukin 1 (IL-1) were observed to influence the transcription of TfR and FT, while their translation was affected by IL-6 and IL-1β [63]. Additionally, cytokines can directly affect the LIP through other means, such as activating IRP (possibly through NO) [64] or liberating iron from unidentified sources like FT or mitochondria [65].

### 2.3. Free Intracellular Iron Accumulation

Iron involvement in ferroptosis was demonstrated when deferoxamine (DFO), an iron chelator, counteracted the deadly effects induced by erastin [19]. Dixon et al. [19] demonstrated that IRP2 controls cellular iron in erastin-induced ferroptosis. Ferritinophagy (autophagic degradation of ferritin) has been associated with ferroptosis by increasing LIP [66]. Degradation of ferritin is mediated by nuclear receptor coactivator 4 (NCOA4), which binds ferritin heavy chain 1 in the autophagosome and delivers it into the lysosome. Knockdown of NCOA4 or blockage of autophagy decreased LIP and ROS production [67], while overexpression of NCOA4 has the opposite effects. Erastin induces ferroptosis by increasing LIP in a time-dependent manner, independent of transferrin entering the cell, and it is blocked by autophagy inhibitor bafilomycin A1 (BafA1) [68]. The study showed that erastin increases ferritin heavy chain 1 (FtH1) synthesis from excess cellular LIP; subsequently, the excess ferritin determines ferritin degradation after ferroptosis induction [68].

One of the three main factors that determines cell death by ferroptosis is an increase in free intracellular iron. Therefore, we will discuss iron accumulation in PD and in diabetes.

#### 2.3.1. Iron Overload in the Brain

The brain requires a considerable amount of energy; therefore, iron is a crucial element for the production of ATP in the mitochondria through the process of electron transport.

Iron plays a crucial role in the synthesis and metabolism of various neurotransmitters in the brain, such as dopamine, norepinephrine, epinephrine and serotonin. Enzymes, such as phenylalanine hydroxylase, tyrosine hydroxylase and tryptophan hydroxylase, are responsible for the synthesis of these monoamine neurotransmitters and are iron-dependent. Furthermore, iron affects other aspects of neurotransmitter metabolism, including uptake, extracellular concentration, interaction with receptors and catabolism. The production of myelin, which is required for nerve conduction, also relies on adequate levels of iron. The oligodendrocytes responsible for producing myelin contain the highest iron concentrations in the brain, as iron is necessary for the synthesis of lipid components of myelin and oligodendrocyte development [69].

Normally, the brain iron concentration is not affected by alterations in the peripheral iron [70]. This is probably because of the blood–brain barrier and the blood–cerebrospinal fluid barrier [71,72]. With age, brain iron accumulation is seen, probably due to increased blood–brain barrier permeability [73].

In the SN of PD patients, iron deposits are present where dopaminergic neurons degenerate selectively, and iron accumulation appears to correlate with disease severity [74,75]. In 1989, the hypothesis was formulated that the iron concentration in SN is the main cause of PD and even named PD “a progressive siderosis of SN” [76].

In SN, iron is mainly bound to ferritin or hemosiderin 2, and less than 15% is bound to neuromelanin [77]. Non-ferritin-bound iron, which is 2000 times lower than the concentration of the total iron, was found to be significantly increased in SNc in PD [78].

The cellular mechanisms responsible for iron accumulation in the brain might be the following:

(1) Alteration of iron export—ferroportin and ceruloplasmin activity are both reduced in PD animal models [70]. Point mutations in ceruloplasmin-encoding genes are associated with PD [79]. Additionally, the levels of amyloid precursor protein (APP) [80], which stabilizes ferroportin on the cell surface and of the soluble tau protein [81], which interacts with APP to help iron export, are both reduced in PD. Rare variants of APP predispose individuals to PD, and APP mutations are associated with PD and Lewy bodies formation [82]. Hepcidin and its mRNA have low levels throughout the brain, suggesting that it might be produced locally [83].
(2) Alteration of iron storage—ferritin and neuromelanin, a veritable alternative “iron well”, are saturated with iron in PD [70]. Both release iron as they become saturated, increasing the LIP [83]. Ferritin is present in SN in microglia, astrocytes, non-pigmented neurons and degenerating dopaminergic neurons.
(3) Increased iron uptake is facilitated by transferrin receptor 2 (TfR2) or the divalent metal transporter 1 (DMT1) or the diffusion of ferric citrate [84]. The Tf/TfR2 combination assists with bringing iron into the mitochondria in SNc neurons [85]. This puts it near the by-products produced during respiration and raises the chances of iron-triggered oxidative stress [86]. Increased vulnerability to PD is linked to the presence of mutated variants of transferrin [87], suggesting that the iron uptake mechanism is overactive in these persons. In contrast, alterations in TfR2, as described by Rhodes et al. [87], were found to have a protective effect against PD. This protective effect could be attributed to a decrease in the ability to uptake functional iron, thereby reducing the risk of elevated iron levels in the body.

The brain, having the second-highest lipid concentration among various organs and a high oxygen consumption rate, is particularly susceptible and sensitive to lipid peroxidation.

#### 2.3.2. Type 2 Diabetes and Iron Overload

The pancreatic islets are highly vulnerable to oxidative damage, possibly due to their heavy dependence on mitochondrial glucose metabolism for insulin secretion in response to glucose stimulation, as well as their limited expression of antioxidant defense systems [88]. Moreover, their elevated expression of the divalent metal transporter makes them more prone to iron accumulation compared to other cells [89], which further increases the risk of iron-mediated oxidative stress.

The initial indication that excessive iron levels in the body could contribute to abnormal glucose metabolism was first observed through the higher occurrence of diabetes in individuals with classic hereditary hemochromatosis [90,91] and those with transfusional iron overload [92,93]. However, as new genetic disorders related to iron metabolism were discovered, it became clear that regardless of the cause or specific genes involved, an elevated iron burden leads to a greater likelihood of developing type 2 diabetes [32].

The involvement of iron in the development of diabetes is supported by two factors: (1) an increased prevalence of type 2 diabetes in various conditions associated with iron overload and (2) the potential for diabetes to improve or even reverse (as indicated by better glycemic control) with the reduction in iron levels through methods such as phlebotomy or iron chelation therapy.

While the precise mechanism by which iron causes diabetes remains unclear, it is probable that three main mechanisms are involved: (1) insufficient production of insulin, (2) reduced responsiveness to insulin (insulin resistance) and (3) impaired liver function [94,95,96].

In a mouse model simulating hemochromatosis, an excessive accumulation of iron and the accompanying oxidative stress induce beta-pancreatic cell death, leading to the decline of pancreatic islets’ ability to secrete insulin [97].

In individuals with type 1 hereditary hemochromatosis, as many as 60% of affected patients develop diabetes [98,99]. Diabetes is believed to arise from a combination of both insulin deficiency and resistance. Iron deposition in the interstitial pancreatic cells, leading to excess collagen deposition and defective microcirculation, leads to insulin deficiency. Evidence supporting this comes from studies conducted on patients with hereditary hemochromatosis, where reducing their body iron stores through methods such as phlebotomy or iron chelation therapy led to improved control of blood sugar levels. In fact, 30–40% of these patients were able to eliminate the need for oral hypoglycemic medication or significantly reduce their dosage [67,99].

An increased incidence of type 2 diabetes is also seen in autosomal dominant hemochromatosis syndrome involving ferroportin, an iron transporter (25% of the patients) [100], and in aceruloplasminemia [101]. Elevated ferritin and transferrin values, even below those seen in hemochromatosis, and iron overload are also associated with abnormal glucose metabolism and insulin resistance in type 2 diabetes [102,103]. These findings provide evidence that iron intake and storage can serve as a marker for identifying type 2 diabetes, potentially enabling early diagnosis in clinical settings [104].

Research conducted on individuals with thalassemia has demonstrated a notable elevation of insulin resistance [93,105]. The mechanisms underlying insulin resistance may involve the potential for iron overload to induce resistance either directly or by impairing liver function [95,96]. Treatment with intravenous or oral chelation therapy has been shown to enhance glucose tolerance in approximately one-third of these patients, indicating that iron may play a causal role in the disease [106,107].

The association between iron overload and improved insulin sensitivity and insulin secretion is indicated by the positive effects observed in individuals who frequently donate blood and consequently have reduced iron stores [108,109].

In 59 to 92% of patients with type 2 diabetes, excess amounts of non-transferrin-bound iron, forming the intracellular LIP, are correlated with the severity of the disease [110].

### 2.4. LIP and Oxidative Stress

#### 2.4.1. ROS Production

ROS (reactive oxygen species) are either free radicals containing oxygen or compounds that are not radicals but have oxidizing properties. Superoxide anions (O_2_^·−^), hydroxyl radicals (OH^·^), lipid radicals (LO^·^, LOO^·^), hydrogen peroxide (H_2_O_2_) and hypochlorous acid (HOCl) are among the most known ROS.

Under normal circumstances, oxygen undergoes complete reduction in the mitochondrial electron transport chain, accepting four electrons and being converted into water. However, in biological systems, partial reduction of oxygen can occur catalyzed by trace amounts of “free” iron from the LIP, producing various ROS, like the superoxide anion, hydrogen peroxide and hydroxyl radicals [111,112]. Mitochondria produce significant quantities of ROS through regular metabolic processes and energy generation within the electron transport chain. Additionally, ROS are naturally formed by xanthine oxidase during the hydroxylation of hypoxanthine to uric acid, catalytic reactions involving cytochrome P450, microsomal processes, NADPH oxidase, cyclooxygenases, uncoupled nitric oxide synthase (NOS), lipoxygenase and peroxisomes, particularly in the liver where fatty acids are oxidized. Furthermore, activated neutrophils, eosinophils and macrophages generate ROS as a defense mechanism against infections [113,114].

In normal, physiological circumstances, the presence of redox-active Fe^2+^ in LIP is carefully regulated to remain at low concentrations of approximately 0.2–0.5 μM, serving essential metabolic requirements [115]. Any surplus Fe^2+^ is securely bound within proteins like ferritin to prevent harmful consequences. Nonetheless, when oxidative stress prevails, elevated levels of superoxide can prompt the release of Fe^2+^ from various iron-containing compounds, including 4Fe–4S clusters, heme and ferritin.

The connection between LIP and ROS production can be intricate due to their reciprocal influences: LIP seems to function not only as a potential source of ROS generation but also as a factor that can be altered by oxidizing or reducing agents impacting cellular iron sources, potentially involving ferritin [116].

Moreover, the production of ROS, independent of iron, might originate from the metabolic breakdown of glucose and glutamine, leading to a reduction in glutathione (GSH) and GPx4 levels [19,117].

While they exhibit moderate reactivity and engage with a restricted set of cellular compounds, ROS are swiftly broken down by antioxidants and specific enzymes like superoxide dismutases (SODs), catalases (CATs), glutathione peroxidases (GPxs) and peroxiredoxins (Prxs). These enzymes are found ubiquitously in cells across all aerobic organisms, despite their relatively low reactivity [118,119,120,121,122].

Increased superoxide production represents only a first step in the production of harmful reactive species that can produce endothelial dysfunction in diabetes. Superoxide overproduction activates protein kinase C (PKC), which induces de novo synthesis of NAD(P)H oxidase. As a result, more superoxide is produced [123]. PKC favors NF-kB activation, which is a transcription factor, and as a result increases the expression of NAD(P)H oxidase and iNOS (inducible nitric oxide synthase). iNOS or NOS2 is one of the three isoforms of NO synthases (NOSs). iNOS produces large quantities of nitric oxide (NO^·^) from L-arginine and oxygen, especially in macrophages, and is induced by proinflammatory conditions [124]. Nitric oxide has a very short biological half-life in vivo (~1 s) and thereby can diffuse a very short distance from its site of birth, within the cell or through the cell membrane [124]. NO^·^ from the endothelial cells diffuses into the nearby smooth muscle cells and binds to the heme iron of guanylyl cyclase, producing cGMP and activating cGMP-cyclic pathways, leading to vasorelaxation [125].

While in normal conditions, nitric oxide is an intracellular messenger that regulates physiological functions, like cardiovascular and neural activities, in pathological conditions NO^·^ can react ultrafast with superoxide, non-enzymatically, to form peroxynitrite (ONOO^−^). NO^·^ can also react with lipid radicals (LO^·^, LOO^·^), resulting LONO and LOONO, and even with OH^·^ [126]. Nitric oxide-derived compounds like nitroxyl (HNO), nitrosonium cation (NO^+^), S-nitrosothiols (RSNOs), peroxynitrite (ONOO^−^) and dinitrogen trioxide (N_2_O_3_) are called reactive nitrogen species (RNS) and are responsible for nitrosative stress. Peroxynitrite is a strong oxidant and cytotoxic because it oxidizes sulfhydryl, iron–sulfur and zinc–thiolate groups in proteins [127] and nitrates tyrosine residues in non-enzymatic reactions [124].

Oxidative stress occurs when the generation of free radicals exceeds their elimination. Free radicals that are not eliminated promptly may lead to the death of dopaminergic cells or the pancreatic beta-cells either by causing peroxidation of lipids in cell membranes or by directly damaging DNA.

#### 2.4.2. ROS and Lipid Peroxidation

Extensive lipid peroxidation of membrane PLs rich in PUFAs is another main factor that determines cell death by ferroptosis.

Normally, GSH binds to iron in the LIP, preventing ferrous iron from being oxidized. This has two important effects: (1) it helps maintain iron solubility, since ferric iron is highly insoluble, and (2) it prevents ferrous iron from acting as a catalyst to produce hydroxyl radicals when there is hydrogen peroxide available in the body. The depletion of GSH leads to the release of iron, which in turn generates hydroxyl radicals and triggers the observed lipid peroxidation in ferroptosis [128].

Lipid peroxidation has been subject to thorough investigation over the last decades. Presently, a widely accepted understanding exists that primary lipid peroxides are formed through the action of hydroxyl radicals generated by Fenton chemistry [129]. Several cell types, including neutrophils, macrophages, neurons and endothelial cells, have the capability to produce both O_2_^·−^ and NO^·^ through distinct actions of various oxidases and nitric oxide synthases [130]. The prominent oxidant within the ONOO^−^/ONOOH system seems to be ONOOH, which swiftly decays through an activated intermediate that shares similarities with HO^·^ [130,131,132].

**Figure 3:**
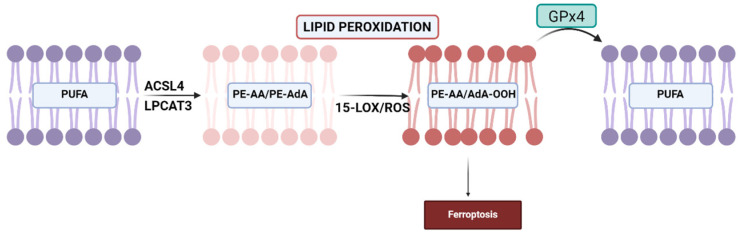
Lipid peroxidation of membranar polyunsaturated fatty acids (PUFAs). ASCL4—acyl-CoA synthase long-chain family member 4; LPCAT3—lysophosphatidylcholine acyltransferase 3; 15-LOX—lipoxygenase 15; PE-AA/PE-AdA—arachidonic acid (AA) or adrenic acid (AdA) containing phosphatidylethanolamine; PE-AA/PE-AdA-OOH—phospholipid hydroperoxides; ROS—reactive oxygen species; GPx4—glutathione peroxidase 4. Created with BioRender.com (accessed on 7 June 2024).

Initially, PUFAs undergo esterification with membrane phospholipids, specifically phosphatidyl ethanolamine (PE). Acyl-CoA synthetase long-chain family member 4 (ACSL4) catalyzes the activation reaction by binding coenzyme A to long-chain PUFAs, enabling their utilization for esterification of lysophospholipids via lysophosphatidylcholine acyltransferase 3 (LPCAT3). The resulting substrates can undergo peroxidation, leading to the formation of arachidonoyl (AA) and adrenoyl (AdA) acids, which can trigger ferroptosis [133,134]. Inhibition of the ACSL4 enzyme suppresses ferroptosis by depleting the substrates required for lipid peroxidation [133,135]. Some studies propose that expression of ACSL4 might serve as an indicator of a cell’s susceptibility to ferroptosis, making it a potential marker [136] (Figure 3).

The oxidation process that culminates in ferroptosis can occur enzymatically or non-enzymatically [137].

(1) Non-enzymatic oxidation involves ROS and hydroxyl radicals generated via the Fenton reaction. This process is non-selective and non-specific, with oxidation rates proportional to the number of readily abstractable bis-allyl hydrogens present in the PUFA molecule. This process is followed by the addition of molecular oxygen, resulting in the formation of the peroxyl radical and the primary molecular product, lipid hydroperoxide (L-OOH). Subsequently, cleavage of the L-OOH molecule occurs, generating highly electrophilic secondary oxidation products such as epoxy, oxo- or aldehyde groups, which are remarkably reactive and detrimental to cell membranes [20]. Consequently, a wide array of oxidation products, primarily oxygenated PUFA-phospholipids (PLs) containing six, five, four, three and two double bonds, accumulates [20,138].

(2) Enzymatic oxidation of PUFAs, on the other hand, occurs through lipoxygenases (LOXs) [68]. LOXs are dioxygenases that contain iron in their catalytic region and facilitate the dioxygenation of polyunsaturated fatty acids possessing at least two isolated cis-double bonds. In humans, multiple isoforms of LOX exist, including 5-LOX, 12S-LOX, 12R-LOX, 15-LOX-1, 15-LOX-2 and eLOX3 [68,138]. Oxygenation by LOX leads to the formation of doubly and triply oxygenated species known as (15-hydroperoxy)-diacylated PE [137]. The oxidation process catalyzed by 15-LOX is selective and specific, with a preference for arachidonic acid–phosphatidylethanolamine (AA-PE) or adrenoyl acid (AdA)-PE. This results in the production of 15-hydroperoxy–arachidonic acid–phosphatidylethanolamines (15-HOO-AA-PEs) or 15-hydroperoxy–adrenoyl acid–phosphatidylethanolamines (15-HOO-AdA-PEs) [137]. 15-LOX catalytic activity depends on phosphatidylethanolamine-binding protein 1 (PEBP1), a pro-ferroptotic protein [139].

Stoyanovsky et al. [140] outlined two stages of the ferroptosis process: (i) the selective and specific enzymatic production of 15-HOO-AA-PE by 15-LOX; and (ii) the oxidative cleavage of these initial hydroperoxide derivatives into electrophiles that can interact with protein targets, leading to the formation of pores in plasma membranes or membrane rupture. The resulting oxidatively truncated products can originate from 15-HOO-AA-PE, with the carbonyl function located either on the shortened AA residue esterified into PE or on the leaving aldehyde.

Vitamin E representatives can effectively inhibit ACSL4, LOX and LPCAT3 enzymes, reducing the build-up of harmful substances that cause cell death [141]. Vitamin E and its esterified analogues (α-tocopherol phosphate and α-tocopherol succinate) compete for the PUFA binding site of the enzymes and, in addition, scavenge hydroxyl radicals. Studies have demonstrated that vitamin E protects cells from ferroptotic death both in vitro [19,22,142] and within GPx4^−^/^−^ knockout mice in living organisms [143,144]. Ferrostatin-1 and liproxstatin-1 prevent ferroptosis by inhibiting lipid peroxidation [22,145], ferrostatin 1 probably acting as iron chelators, due to its structure.

Recently, Zou et al. [146] identified another contributor for ferroptotic cell death, the POR (cytochrome P450 reductase). “POR may facilitate lipid peroxidation by accelerating the cycling between Fe (II) and Fe (III) in the heme component of CYPs (cytochrome P450)”.

#### 2.4.3. ROS and DNA Damage

Hydroxyl radicals can attack the sugar–phosphate bonds in DNA by abstracting hydrogen atoms from the deoxyribose or can introduce -OH groups to the double bonds of the pyrimidine and purine rings [147]. Since the number of iron ions is limited in the nucleus (a small LIP pool demonstrated by Petrat et al. [148]), it seems that multiple cycles of oxidations and reductions of iron ions occur at the same iron center [149]. A substantial correlation between the cellular LIP level and the production of 8-oxodGuo, a typical marker of ROS-induced DNA damage, was observed in human lymphocytes [150] and with DNA breaks, in H_2_O_2_-treated cells [43]. The sequestering of intracellular iron with desferrioxamine (otherwise known as deferoxamine or desferal) prevents the occurrence of DNA strand breaks and the cytotoxic effects induced by H_2_O_2_ on both nuclear [151,152] and mitochondrial [153] DNA. Enhancing the expression of the heavy subunit of ferritin (FtH) led to a reduction in LIP, resulting in diminished responses to oxidative stress [154] and protected cells against H_2_O_2_-triggered cytotoxicity and genotoxicity [48]. Conversely, reducing the cellular FT level by inhibiting its synthesis through antisense techniques resulted in elevated LIP levels [155] and heightened vulnerability to oxidative stress [156].

### 2.5. Antioxidant Systems Involved in Ferroptosis

Another main factor determining cell death through ferroptosis is the depletion of the antioxidant systems that remove ROS and control oxidative stress. We will discuss three such antioxidant systems and their involvement in PD and T2D.

#### 2.5.1. GSH, GPx4 and System xc^−^ Depletion

The most well-known mechanism that suppresses ferroptosis involves GPx4 [157]. Glutathione peroxidases catalyze the reduction of H_2_O_2_ and organic/inorganic hydroperoxides to water or their corresponding alcohols using GSH.

Among the eight glutathione peroxidases present in mammals, which vary upon tissue expression, cell location, structure and substrate specificity [158], GPx4 (originally known as phospholipid hydroperoxide glutathione peroxidase, PHGPx) emerges as a particularly vital component within the GSH system and is one of the most studied and most important enzymes in the ferroptotic process [159]. GPx4 is the only enzyme that can reduce complex hydroperoxy ester lipids such as hydroperoxy phospholipids and hydroperoxy cholesterolesters, even when they are still located in the biological membrane [120,160,161], as well as thymine hydroperoxide, a product of free radical attack on DNA [162].

**Figure 4:**
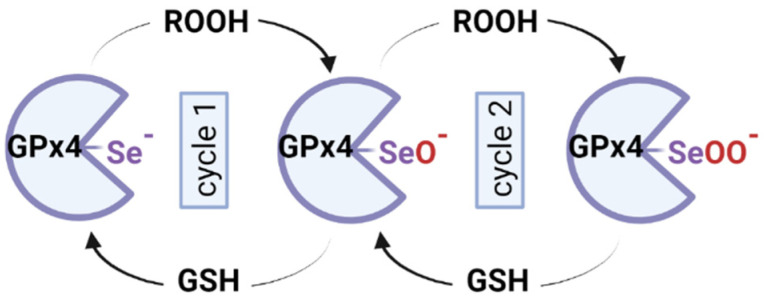
Different redox states of the selenocysteine residue from the catalytic site of GPx4. Created with BioRender.com (accessed on 6 June 2024).

GPx4 is encoded by the GPx4 gene in chromosome 19 (band 19p13.3), is composed of 197 amino acids and has a 20–21 kDa weight [163]. A selenocysteine residue (Sec46) in the active site of GPx4 is essential for its activity and is also the site of RSL3 binding [68]. The selenocysteine involves three different redox states: selenol (Se-H), selenenic acid (Se-OH) and seleninic acid (Se-OOH), allowing the regulation of the catalytic efficiency depending on the cellular redox state [160] (Figure 4).

The GPx4 has a typical thioredoxin motif containing four α-helices located near the protein surface and seven β-stands, five of them in the central region [160]. Near Sec46, there are three more amino acids (forming a catalytic tetrad), observed also in other GPxs: glutamine (Gln81) and tryptophan (Trp136), which stabilize the selenium redox intermediates (Se, SeO^−^), and asparagine (Asp137), which has a role in the catalytic activity [164]. Mutations of selenocysteine with cysteine reduce its activity by 90%, and mutations of any of the other three amino acids greatly diminish its activity [165].

GPx4 is present as three physiological isoforms: cytosolic (cGPx4), mitochondrial (mGPx4) and nuclear (nGPx4) [166]. mGPx4 protects the cell from mitochondrial ROS generated in the respiratory chain, the pyruvate dehydrogenase complexes and other α-keto acid dehydrogenases [167]. Only cGPx4 is required for ferroptosis protection. In the liver, lipid stress induces the expression of a transcript variant of GPx4, iGPx4, which is keeping GPx4 in its oligomeric, inactive form, thus promoting oxidative stress and ferroptosis [168].

**Figure 5:**
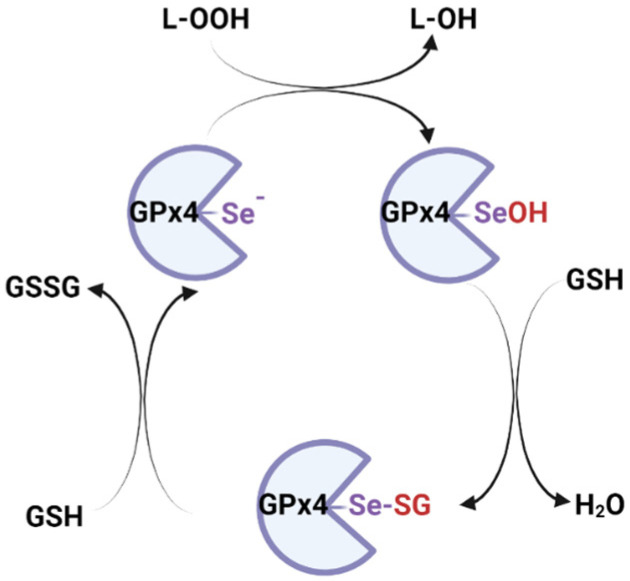
The GPx4 catalytic cycle. Adapted from [170]. Created with BioRender.com (accessed on 6 June 2024).

GSH is used as the preferred cofactor for mammalian enzymes, but GPx4 has the unique ability to use other protein thiols, too [169]. The catalytic cycle has two different stages, following a ping-pong mechanism: the first stage in which the peroxides are reduced concomitantly with the oxidation of selenol (Se-H) to selenenic acid (Se-OH) and a second stage where the selenenic acid is reduced back to selenol, using two GSH (Figure 5).

GPx4-Se^−^+ L-OOH → GPx4-SeOH + L-OH

GPx4-SeO^−^ + H^+^ + GSH → GPx4-Se-SG + H_2_O

GPx4-Se-SG + GSH → GPx4-Se^−^ + H^+^ + GSSG

A seleninic acid (Se-OOH) has also been observed in the active site of the crystalized enzyme, suggesting that GPx4 can go through low or high oxidation cycles of selenol, depending on the oxidative stress status of the cell [165].

**Figure 6:**
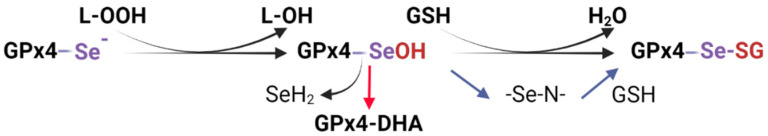
Alternative reactions of GPx4 associated with high levels of lipid peroxides. Adapted from [170]. Created with BioRender.com (accessed on 6 June 2024).

It seems that at high levels of lipid peroxides associated with low GSH levels, GPx4 loses Se through β-cleavage of selenenic acid and forms dehydroalanine (DHA), an enzymatic dead form of the enzyme. As a protective mechanism, selenenic acid can form an intermediate with the amino group of the adjacent glycine, selenylamide (-Se-N-), until the cellular redox conditions are restored [170] (Figure 6).

In PD, plasma selenium is decreased [171]. Se is present in higher concentrations in SN and putamen than in other brain regions [172]. Several selenoproteins, besides GPx4, are present in the brain (SELENOP, SELENOT) protecting dopaminergic neurons against oxidative stress [172]. Alim et al. [173] demonstrated that pharmacological Se supplementation (usually as sodium selenite) drives GPx4 and other antioxidant selenoproteins expressions to counter ferroptosis and protect neurons. The amount of free selenium delivered in the cell determines the sensitivity to ferroptosis in any tissue, by transcriptional regulation of selenoproteins. They developed a peptide (Tat SelPep) that transports selenium and delivers it for GPx4 synthesis across the blood–brain barrier and in the heart and liver. This peptide contains a transduction domain from the HIV-Tat protein that links covalently and delivers several cargoes, attached to six amino acids from the C-terminal domain of SELENOP, which includes multiple selenocysteine residues. Tat SelPep protects neurons from hemin-induced and homocysteic acid-induced ferroptotic cell death in vitro and in vivo. It might be used to supplement selenium in the whole body and ameliorate neurodegenerative diseases linked to GPx4 disfunction. In contrast, agents that suppress GPx4 transcription and other genes of the selenome might be used to kill tumors by ferroptosis [117,170,173].

Increased plasma SELENOP levels are also seen in type 2 diabetes because its biosynthesis is increased by high glucose concentrations and is suppressed by insulin [172].

GSH availability depends on system xc^−^, a heterodimeric transmembrane antiport complex. The xc^−^ system consists of a heavy chain, solute carrier family 3 member 2 (SLC3A2) and a light chain, solute carrier family 7 member 11 (SLC7A11/xCT) and helps cystine enter the cell. Cystine is then reduced to cysteine either by intracellular GSH or by thioredoxin reductase 1 (TRR1), which is mainly used for GSH synthesis [137].

GSH synthesis is regulated by nuclear factor erythroid 2-related factor 2 (Nrf2) [174]. In basal conditions, Nrf2 activity is suppressed by Keap1 (kelch-like ECH-associated protein 1)-controlled ubiquitination–proteasomal degradation. Nrf2 is activated by various stimuli, including oxidative stress, due to modification of critical cysteine thiols of Keap 1 and Nrf2, leading to nuclear accumulation of activated Nrf2. Active Nrf2 mediates induced expression of GSH biosynthesis enzymes: glutamate cysteine ligase (GCL), GSH synthase, GSH S-transferases (GSTs), GSH reductase (GR) and the light-chain subunit of the xc^−^ system [137]. There are several studies that support the protective role of Nrf2 against neurodegenerative diseases, leading to new possibilities for drug development [175]. Nrf2 inhibitors might be developed to treat cancers that have elevated Nrf2 activities, as cancer cells could exploit Nrf2’s protective ability to boost their own growth and resistance to drugs.

It appears evident that the initiation of ferroptosis primarily occurs due to a decrease in the capability to detoxify lipid peroxides through the enzymatic function of GPx4 or due to the absence of this ability because of GSH depletion or lack of GSH synthesis (system xc^−^ inhibitors) [142]. RAS-selective lethal small molecule 3 (RSL3) directly inactivates GPx4, while erastin acts indirectly [17,176]. Erastin depletes its reducing substrate GSH by blocking system xc^−^, which provides cysteine for GSH synthesis. Additionally, inhibitors of SLC7A11 include sorafenib and sulfasalazine. Various other inducers of ferroptosis encompass buthionine sulfoximine (BSO) [22], which is an irreversible inhibitor of γ-glutamyl cysteine synthase, the rate-limiting enzyme of GSH synthesis, as well as pharmacological agents like acetaminophen, lanperisone and artesunate [24].

From a metabolic perspective, when there is a deficiency of cysteine, enhanced glutaminolysis, fueled by an abundance of L-glutamine, promotes ferroptosis [177]. Moreover, mutant p53, by inhibiting the transcription of the cystine glutamate antiporter (SLC7A11), can amplify ferroptosis [178]. Notably, some mutant variants of p53 might not suppress SLC7A11 expression. For instance, a mutated p53 in the gene’s N-terminal domain loses its functionality in reducing SLC7A11 [179].

NADPH oxidase promotes ferroptosis by increasing the generation of ROS and inhibiting the expression of SLC7A11. Additionally, ferroptosis can be induced by compounds like holotransferrin (transferrin in its iron-bound form) [114].

These findings suggest that GPx4 is a crucial regulator of ferroptosis and a promising therapeutic target for degenerative diseases and diabetes.

#### 2.5.2. NAD(P)H/FSP1/CoQ System

**Figure 7:**
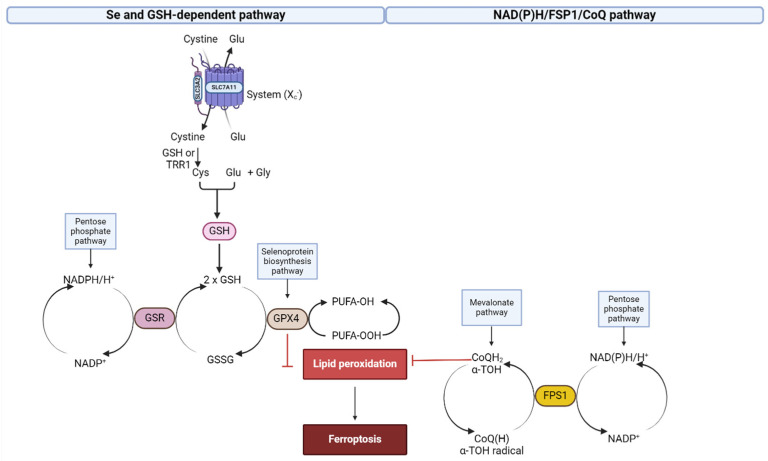
Two metabolic pathways controlling ferroptosis. Adapted from [170]. Created with BioRender.com (accessed on 6 June 2024).

Doll et al. [180] described a parallel standalone system that suppresses lipid peroxidation and ferroptosis and co-operates with the GPx4 and GSH system (Figure 7). Inhibition of GPx4 fails to trigger ferroptosis in some cancer cell lines, and cells proliferate indefinitely. Ferroptosis suppressor protein 1 (FSP1) functions independently of cellular GSH levels, GPx4 activity or ACSL4 expression and oxidizable fatty acid content. It functions as a NAD(P)H-dependent CoQ oxidoreductase in vitro [181].

FSP1 is found in the outer mitochondrial membrane, the ER and the plasma membrane. Only FSP1 found in the plasma membrane has a short N-terminal hydrophobic sequence which is myristoylated, which permits its recruitment to the plasma membrane, and only this enzyme is necessary and sufficient for ferroptosis inhibition [182].

Doll et al. [180] data suggested that FSP1 selectively sensitizes cells to ferroptosis inducers, while GPx4 knockout tumors grow normally. The effect of FSP1 is mediated by coenzyme Q (CoQ). The reduced form of CoQ, ubiquinol, has been reported to be a lipid peroxyl radical scavenger in the mitochondria, but its role outside the mitochondria is considered to be limited, because a recycling mechanism has not been described [183]. FSP1 catalyzes the regeneration of CoQ using NAD(P)H as a cofactor, meaning that the reducing equivalents from NAD(P)H are transferred into the lipid bilayer through CoQ and stabilize lipid peroxyl radicals, inhibiting propagation of lipid peroxidation. The same effect had α-tocopherol administration which can be regenerated either by reduced CoQ or by FPS1 directly. Their results suggested that the ferroptosis sensibility can be predicted by the NAD(P)H and CoQ levels and that combining GPx4 inhibitors with FSP1 inhibitors might be a better cocktail for ferroptosis induction in therapy-resistant cancer cells [180].

In 2016, FIN56, a synthetic compound, was found to induce ferroptosis, leading to cell death by depleting ubiquinone. FIN56 alters the mevalonate pathway, upstream of ubiquinone synthesis, activating squalene synthase (SQS), an enzyme involved in cholesterol synthesis. Therefore, farnesyl pyrophosphate (FPP) becomes scarce for the synthesis of other biomolecules such as vitamin K and CoQ. Supplementation with FPP, idebenone, a soluble analogue of ubiquinone, and other non-steroidogenic products of the mevalonate pathway suppresses FIN56 lethality [184].

Dimethylallyl pyrophosphate (DMAPP), an intermediate in the mevalonate pathway, is also involved in GPx4 synthesis. For selenocysteine’s introduction in its active site, a specific tRNA [Ser] Sec is necessary [185]. This tRNA is one of the longest tRNA in mammals (90 nucleotides) and contains a few modified bases, including i6A37 (N6-isopentenyl-adenosine-37). The isopentenylation is accomplished by Trit1 enzyme, a dimethylallyl/tRNA[Ser]Sec transferase, and is essential for full expression of GPx4 [186]. It was suggested by Shimada et al. [184] that the decreased expression of GPx4 observed by FIN56 treatment was due to its interference with the isopentenylation reaction of tRNA [Ser]Sec.

Because the mevalonate pathway is involved in producing intermediates necessary for both CoQ and GPx4 synthesis, it would be interesting to explore the impact of statins and other mevalonate-blocking drugs on ferroptosis-related diseases. Overexpression of FSP1 needs further investigations to prove its efficiency in protecting neurons from ferroptosis in Parkinson’s disease.

#### 2.5.3. GCH1/BH_4_/DHFR System

**Figure 8:**
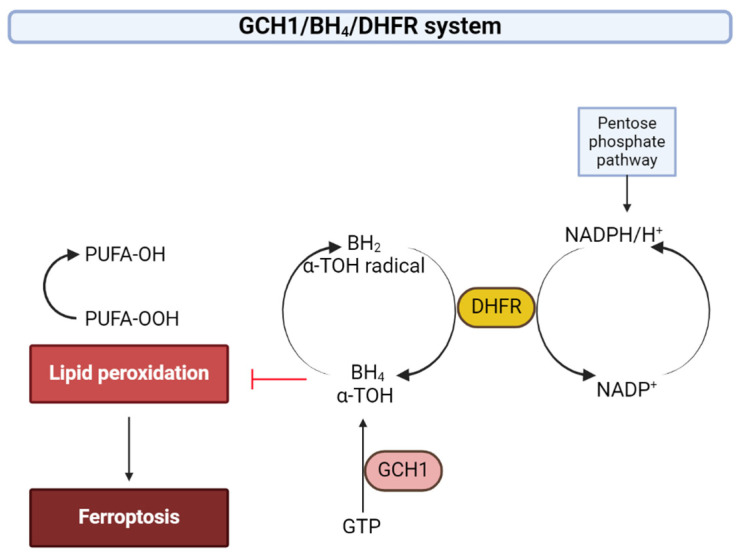
The GCH1/BH_4_/DHFR system controlling ferroptosis. Created with BioRender.com (accessed on 7 June 2024).

Another antioxidant produced endogenously, tetrahydrobiopterin/dihydrobiopterin (BH_4_/BH_2_), was recently identified by genome-wide CRISP/Cas-mediated activator screen by Kraft et al. to suppress ferroptosis [187]. BH_4_ is a cofactor for iron-containing enzymes to produce aromatic amino acids, neurotransmitters and nitric oxide. Reduced BH_4_ availability determines reduced NO^·^ productions by nitric oxide synthases (NOSs) and increased endothelial superoxide production due to enzymatic “uncoupling” of eNOS, at the expense of NADPH [188]. Superoxide production could lead to subsequent reactions, generating H_2_O_2_ and hydroxyl radicals. If some NO^·^ is in the vicinity, peroxynitrite anion (ONOO^−^) can be generated, contributing to oxidative/nitrosative stress. Increased oxidative stress leads to further BH_4_ oxidation into BH_2_ or biopterin [188]. Dihydrofolate reductase (DHFR) can “recycle” BH_2_ and thus regenerate BH_4_ with the help of NADPH (Figure 8). BH_2_, which has no cofactor activity, binds to eNOS with the same affinity as BH_4_ [189]. If BH_2_ binds to eNOS, eNOS uncoupling occurs and superoxide is produced. BH_4_ levels are thus determined by its de novo synthesis, its oxidation to BH_2_ and its regeneration by DHFR (the so-called “salvage pathway”). The eNOS uncoupling is now believed to be determined not by the concentrations of BH_4_ but rather by the BH_4_/BH_2_ ratio [190]. Dihydropteridine reductase (DHPR) is essential for BH_4_ regeneration in tissues where its presence is necessary for the activity of the aromatic amino acid hydroxylases (phenylalanine, tyrosine and tryptophan hydroxylases) and not for eNOS, like the liver and the neurons [189]. In fact, in the brain, DHFR concentration is relatively low [191].

BH_4_ acts directly as an antioxidant, selectively preventing oxidation of phospholipids having two unsaturated acyl chains [187].

BH_4_ deficiency affects dopamine synthesis (because it is the cofactor of tyrosine hydroxylase) in dopaminergic neurons in the nigrostriatal system, which leads to neuron degeneration and has been implicated in movement disorders in PD. The neurodegeneration, severe motor defects and premature death are directly proportional with the extent of BH_4_ deficiency (shown in familial PD caused by GCH1 gene mutations). BH_4_ is also involved in maintaining mitochondria health, which means that reduced levels of BH_4_ are associated with impaired firing of dopaminergic neurons that release dopamine [192].

Additionally, BH_4_ can influence CoQ levels by converting phenylalanine to tyrosine, which can be further transformed into 4-OH-benzoate, a precursor for CoQ [187].

BH_4_’s binding to eNOS changes the conformation of the enzyme, increasing its affinity for binding arginine, and plays a role in dimer formation of the active enzyme [189,193]. De novo synthesis of BH_4_ is impaired due to low GTP cyclohydrolase-1 (GCH1) activity in diabetes and high glucose state [194]. BH_4_ is oxidized into BH_2_ in diabetes and leads to eNOS disfunction due to eNOS uncoupling. Dysfunctional eNOS determines decreased muscle blood flow and glucose uptake, which leads to insulin resistance. In the liver, BH_4_ lowers blood glucose by glucose uptake and by suppressing hepatic gluconeogenesis in an eNOS-dependent manner and ameliorates glucose intolerance and insulin resistance in diabetic mice [194].

The rate-limiting enzyme in BH_4_ synthesis is GTP cyclohydrolase-1 (GCH1 or GTPCH). GCH1 alterations have been observed in chronic diseases such as diabetes [188,195], arthrosclerosis and hypertension and are implicated in PD [196,197]. Nuclear factor erythroid 2-related factor 2 (Nrf2) stimulates GCH1 expression in cells transfected with Nrf2 [198]. GFRP (GCH1 feedback regulatory protein) forms a 2:1 complex with GCH1, leading to enzyme inhibition/activation, depending on cofactor availability—phenylalanine acts as an allosteric activator while BH_4_ and BH_2_ act as allosteric inhibitors. In oxidative stress, the GFRP expression is enhanced, leading to a decrease in BH_4_ levels [199].

GCH1 overexpression yields almost complete protection against ferroptosis induced by RSL3 and imidazole ketone erastin (IKE) [187] and BH_2_ or BH_4_ supplementation increases the survival rate of the cells [187,200].

Mencacci et al. [201] showed that rare GHC1 variants need to be considered as risk factors for developing PD and that aside from dopamine depletion, GCH1 deficiency leads to nigrostriatal degeneration.

Cronin et al. [192] showed that loss of dopaminergic neurons depends on the extent of BH_4_ deficiency; BH_4_ is also used for maintaining energy and ROS levels in mitochondria and thus the firing rates of dopaminergic neurons.

There is a growing interest into developing drugs that target GCH1. The enzyme is found in cytosol, which makes it is easier to target than enzymes located in the nucleus or mitochondria, but its complex structure makes it difficult to design drugs that cannot affect other proteins. ProSavin and OXB-102 are under investigation in clinical trials using lentivectors; ProSavin showed long-term safety and tolerability and motor symptom improvement in PD [202].

Thus, BH_4_ is a good target for treating the vascular complications of diabetes as well as diabetes itself, and activation of the recycling pathways may be one way, along with BH_4_ supplementation, and reducing oxidative stress with various agents.

### 2.6. Toxic Iron Interactions in PD

In PD, iron can interact with specific components within the dopaminergic neurons, leading to more toxic species that can harm these neurons.

#### 2.6.1. Iron and Neuromelanin (NM)—A Toxic Couple in PD

NM is a pigment composed of different components: melanin, lipids and metal ions (the most abundant is iron, followed by zinc, copper and others in lower amounts) and xenobiotics from cytosol, and it is present in cytoplasmic special autolysosomes. It exists in different neurons of the brain and is particularly abundant in dopamine (DA) neurons of the SN and locus coeruleus [203], accumulating continuously with age. NM is formed in SNpc neurons in the first 3 years from birth and increase linearly with age [204].

NM is the primary molecule for storing iron in the dopaminergic neurons of the human SN. This fact has been demonstrated in studies by Zucca et al. [205] and is of particular interest. Magnetic resonance imaging (MRI) studies have revealed that iron builds up in the early stages of PD, even before symptoms appear [206,207,208].

Following the synthesis of DA, the vesicular monoamine transporter 2 (VMAT2) facilitates the transportation of DA from the cytosol into synaptic vesicles. This process prevents the accumulation of DA in the cytosol and the subsequent formation of neurotoxic substances [209,210]. The enzymatic degradation of cytosolic DA also plays a crucial role in safeguarding dopaminergic neurons from neurotoxicity.

If DA is not sequestered within synaptic vesicles, its enzymatic degradation prevents the accumulation of DA in the cytosol. DA degradation starts with a deamination catalyzed by monoamine oxidases (MAOs) located in the outer mitochondrial membrane, generating 3,4-dihydroxyphenylacetaldehide (DOPAL) [210]. Normally, DOPAL is transformed into less neurotoxic 3,4-dihydroxyphenylacetic acid (DOPAC) by aldehyde dehydrogenase (ALDH) and a minor metabolite, 3,4-dyhydroxyphenylethanol (DOPET), via aldehyde/aldose reductase. ALDH inhibition or vesicular uptake increases endogenous DOPAL levels [211]. ALDH enzymes are sensitive to oxidative stress and lipid peroxidation, both elevated in PD [212,213]. Continuous production of DOPAL occurs in dopamine neurons due to several factors. These factors include the following: ongoing DA synthesis in the cytosol, leakage of DA from vesicular stores into the cytosol, incomplete recycling of DA from the cytosol back into the vesicles and reuptake of DA released through exocytosis [210].

DOPAL is toxic to dopaminergic neurons, both in vitro and in vivo, exerting its toxicity by protein cross-linking, oxidation to quinones, production of hydroxyl radicals, augmenting other agents’ toxicity and precipitating alpha-synuclein (α-Syn) aggregates in neurons [210]. In vitro studies have demonstrated that oligomers formed by the combination of α-Syn and DOPAL can disrupt the integrity of synaptic vesicles, resulting in the leakage of DA and exacerbating the cycle of DOPAL generation [214].

The formation of NM, an end-stage product of DA metabolism in the SNc, involves the formation of DA complexes with ferric iron. These complexes undergo a redox reaction with ferric iron being reduced to ferrous iron, and DA-o-quinones are obtained (i.e., DA-o-quinone, aminochrome, indole-quinone) [69,215]. These quinones are not stable in cytosol and undergo modifications into more stable forms (5,6-dihydroxyindole, 5,6-indolequinone) [69]. Quinones react with specific amino acids such as cysteine, histidine and lysine within fibrillar proteins, which serve as the seeds for the formation of aggregated oligomers of peptides or proteins. Subsequent oxidation leads to the production of melanin. Among the proteins capable of interacting with quinones, α-Syn has been identified in the NM-containing organelles of the SN. However, it is likely that other proteins with cross-beta structure are also involved in the formation of insoluble fibrils. The aggregates are resistant to degradation by the proteasome system. As a result, they become incorporated into double-membraned autophagosomes, which subsequently fuse with lysosomes to form autolysosomes. These autolysosomes further fuse with other vesicles containing additional proteins and lipids. Ultimately, within the autolysosomes, the adducts of iron–melanin–beta sheet proteins react with lipids to generate the final neuromelanin (NM) pigment within the organelle [69,216].

The DA-o-quinones obtained induce mitochondria dysfunction by forming adducts with proteins from complexes I, III and V of the electron transport chain and oxidative phosphorylation [217]. They also form adducts with proteins involved in protein degradation and processing, inactivating the proteasomal system [218].

This pathological mechanism can sustain itself, as research has demonstrated that certain quinones, such as aminochrome, can alter the expression of proteins involved in regulating iron balance. Specifically, they increase the levels of DMT1 and decrease the expression of ferroportin [219]. Consequently, this leads to an enhanced accumulation of iron and potentially promotes further oxidation of DA.

NM biosynthesis depends on cytosolic concentration of DA not sequestrated in synaptic vesicles, so it seems to be a protective process that removes excess of cytosolic catechols (DA, norepinephrine and their metabolites) [220]. Besides that, it can sequester reactive/toxic compounds, metal ions and different xenobiotics from the cytosol, effectively immobilizing them by forming stable adducts.

Surprisingly, in PD, the NM-containing neurons are most vulnerable to destruction, and severe depigmentation is observed [221]. In PD and parkinsonian syndromes, the pigmented dopaminergic neurons in the SN undergo degeneration, resulting in the release of a significant amount of accumulated NM into the extracellular space. The organelles containing NM, left behind by dying neurons, release the previously accumulated reactive/toxic metals and organic toxins into the surrounding environment. Microglial phagocytosis and NM degradation also release toxic compounds. Activated microglia contribute to toxicity by producing and releasing reactive species such as H_2_O_2_ and peroxynitrite, as well as proinflammatory cytokines that further exacerbate microglial-mediated toxicity [222]. Evidence suggests also the occurrence of reactive astrocytosis around the deposits of NM [223]. The presence of both reactive astrocytes and microglia indicates a significant level of inflammation within the SN of the brain affected by PD [223,224,225,226].

#### 2.6.2. Alpha-Synuclein–Iron Couple in PD

α-Syn is the main protein component of the intracellular inclusions known as Lewy bodies in sporadic PD patients’ postmortem brain tissue [227]. α-Syn is a protein composed of 140 amino acids, encoded by SNCA gene highly expressed in the nervous system, particularly near the presynaptic membrane, in close proximity to the synaptic vesicles, suggesting its involvement in neurotransmission [228]. This role has been confirmed in SN axons [204].

α-Syn is involved in the assembly of the SNARE complex (soluble N-ethylmaleimide sensitive-factor attachment receptors) functioning as a chaperon-like protein [228]. SNARE complexes mediate the fusion of vesicles with the target membranes; thus, α-Syn controls neurotransmitter release [228]. Additionally, α-Syn can undergo conformational changes, forming insoluble fibrils through various intermediate oligomeric states [229,230]. Research indicates that these soluble oligomers and prefibrillar species are highly toxic to cells, rather than the fibrils themselves. The formation of Lewy body inclusions, characterized by α-Syn aggregates, may contribute to neuronal cell death by interacting pathogenically with lipids, organelles and membrane structures [231,232].

The oxidation by-products of DA and other catecholamines can interact with α-Syn, resulting in the formation of modified α-Syn that is less prone to fibrilization and instead forms soluble oligomers [233,234,235,236]. Studies using fluorescence-lifetime imaging microscopy have revealed that DA induces a conformational change in α-Syn, bringing the N- and C-terminals closer together, potentially inhibiting fibril formation. Additionally, the DA metabolite DOPAL can cross-link lysine residues in α-Syn, facilitating its aggregation [237].

Intracellular oligomeric forms of α-Syn can be cytotoxic through various mechanisms, including the formation of pore-forming fibrils that permeabilize vesicular and plasma membranes, disruption of proteasomal protein clearance, chronic endoplasmic reticulum stress, mitochondrial dysfunction and inhibition of SNARE complex formation and neurotransmitter release [204].

α-Syn has been observed within the NM-organelles, likely resulting from the reaction of fibrillar α-Syn with DOPAL during the early stages of NM synthesis, forming a DOPAL-α-Syn adduct that is subsequently oxidized into a non-toxic melanin–protein complex. This complex is then engulfed by autophagic vacuoles, which undergo further processing to generate the final NM-organelles [216].

α-Syn undergoes various post-translational modifications, including oxidation and nitration, which are triggered by an excess of iron and iron-induced oxidative stress. Both ferrous [238] and ferric [239] forms of iron directly bind to the C-terminus of α-Syn [240,241]. The interaction between α-Syn and iron promotes its aggregation into fibrils by inducing conformational changes [238,242,243] and facilitates the oligomerization of α-Syn [244,245]. The ability of α-Syn to bind iron is crucial for its pathological aggregation processes. Alternatively, it has been proposed that α-Syn functions as a ferrireductase, maintaining the equilibrium between ferrous and ferric iron in normal physiological conditions [241].

In the 5′ untranslated region (5′UTR) of the α-Syn mRNA it has been identified a possible IRE. This implies that an increase in iron levels could potentially enhance the translation of α-Syn by binding to the IRE. Supporting this notion, it has been observed that depletion of iron leads to a decrease in the translation of α-Syn [246].

Inhibition of transferrin receptor (TfR) prevented the overexpression and aggregation of α-Syn in response to 1-methyl-4-phenylpyridinium (MPP^+^) treatment in neuroblastomas [247]. Cerebral ceruloplasmin (CP), which plays a role in iron metabolism, potentially co-localizes with α-Syn in Lewy bodies (LBs) and could induce the aggregation of α-Syn [248]. These findings indicate a direct interaction between iron and α-Syn, suggesting a potential convergence of the two in a shared pathological pathway in PD, such as ferroptosis.

### 2.7. Oxidative Stress and Glucose Toxicity in PD and T2D

Most cells can decrease the influx of glucose into the cell in response to high blood glucose levels, ensuring that the glucose concentration inside the cell remains constant. In diabetes, capillary endothelial cells in the retina, neurons and Schwann cells in the peripheral nerves and mesangial cells in the renal glomerulus cannot do this, and they can be damaged by hyperglycemia [249].

**Figure 9:**
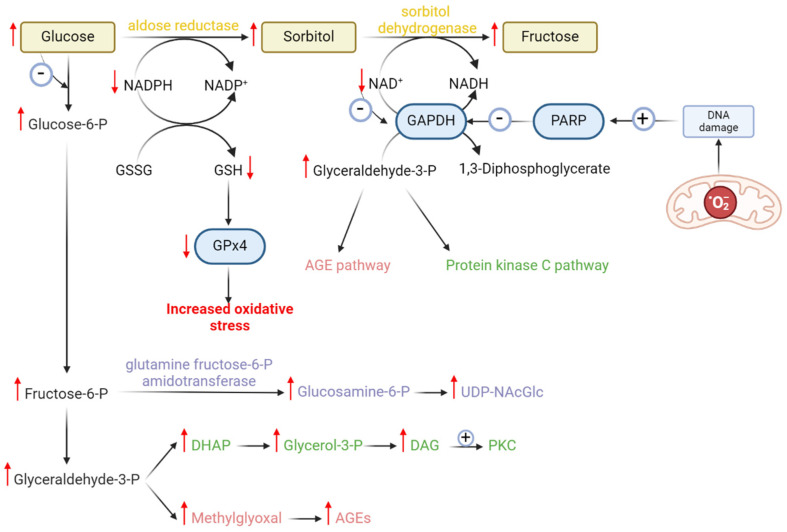
Unifying mechanism of hyperglycemia-induced damage. In yellow—polyol pathway; in red—AGEs production; in green—PKC activation; in purple—hexosamine pathway. Created with BioRender.com (accessed on 8 June 2024).

Four molecular mechanisms seem to be involved in tissue damage caused by hyperglycemia: (1) increased polyol pathway flux, (2) increased advanced glycation end products (AGE) production, (3) protein kinase C activation and (4) increased hexosamine pathway activity (Figure 9) [249,250].

It is widely agreed upon that oxidative stress is likely the main factor that causes diabetic complications induced by hyperglycemia, according to various sources [249,251,252].

#### 2.7.1. Increased Polyol Pathway Flux

In hyperglycemic conditions, hexokinase is substrate-inhibited, and glucose must be consumed by other pathways. At a normal glucose concentration, only a small part of glucose is fueled into the polyol pathway because aldose reductase has a lower affinity for glucose than hexokinase [253]. Aldose reductase functions normally to reduce toxic aldehydes derived from ROS involvement to inactive alcohols. It is found in the nerves, retina, lens, glomerulus and vascular cells [254]. When intraneuronal glucose concentration is elevated, aldose reductase transforms the increased flux of glucose in fructose using both NADPH, derived from the pentose phosphate pathway, and NAD^+^. NADPH consumption is likely to lower GSH regeneration, which is most necessary to maintain glutathione peroxidase activity, thus increasing the susceptibility to intracellular oxidative stress (Figure 9) [255]. Superoxide overproduction significantly inhibits glucose-6-phosphate dehydrogenase, the key enzyme in the pentose phosphate pathway, enhancing NADPH loss [256].

NAD^+^ concentration is decreased, both by its usage in the polyol pathway and in the repair process, and that inhibits the glyceraldehyde 3-phosphate dehydrogenase (GAPDH) and therefore the glycolysis [256]. GAPDH inhibition determines the accumulation of upstream triose phosphates which can lead to the synthesis of methylglyoxal, a precursor of the advanced glycation end-products (AGEs) [257]. Glyoxalase uses GSH to deactivate methylglyoxal, which further depletes its cellular levels [258].

#### 2.7.2. Increased AGE Production

AGEs formation starts with non-enzymatic condensation between the carbonyl groups of reducing sugars and the free amino groups of nucleic acids, proteins or lipids. This process is followed by further rearrangements resulting in reactive carbonyls such as glyoxal, methylglyoxal, glyceraldehyde, glycoaldehyde, diacetyl and 1- and 3-deoxyglucosone. Reactive carbonyls are also generated from glycerldehide-3-phosphate obtained from glycolysis or from fructose 1-phosphate obtained from fructolysis or glucose oxidation. Further condensation with the available amine groups yields a great variety of final AGEs, which are classified by their precursor (Figure 9) [259]. In healthy people, glycated plasma proteins levels are below 3% [260]. However, in conditions like diabetes, these levels can increase up to threefold, leading to the development of a pathological phenotype [259].

AGEs are believed to play a role in the development of the major microvascular complications of diabetes mellitus, which include nephropathy, neuropathy and retinopathy [250]. Besides modification of intracellular proteins by glycation, including proteins involved in the regulation of gene transcription [261,262], AGEs precursors can diffuse out of the cell, modifying extracellular matrix molecules and leading to cellular dysfunction because of changes in signaling between the matrix and the cell [249]. They can also modify circulating proteins that can bind to AGE receptors, causing ROS production and later inflammatory cytokines and growth factor production leading to vascular pathology [249]. Also, one of the most important enzymes involved in ROS defense mechanism, SOD, is fragmented after glycation [251].

#### 2.7.3. Protein Kinase C Activation

Hyperglycemia activates classic isoforms of protein kinase C (PKC) β, δ and α through an increase in de novo synthesis of diacylglycerol, an activating factor for the enzymes [249]. Overproduction of mitochondrial superoxide also initiates de novo synthesis of diacylglycerol (DAG) or phosphatidylcholine hydrolysis that activates PKC (Figure 9) [250,256]. PKC activates NAD(P)H oxidases, which produces more superoxide [251], and it influences gene expression in various ways. PKC also activates NFkB, a transcription factor that, in turn, activates proinflammatory genes within the vascular system [256]. Brownlee says: “The things that are good for normal function are decreased and the things that are bad are increased” [249]. As a result, blood flow abnormalities, capillary and vascular occlusion, vascular permeability modifications and production of inflammatory cytokines and growth factors happen.

#### 2.7.4. Increased Hexosamine Pathway Activity

While under metabolic conditions, only 2–5% glucose is directed to the hexosamine pathway [250], and due to overproduction of superoxide caused by hyperglycemia, glyceraldehyde 3-phoshate dehydrogenase (the enzyme from glycolysis) is inhibited, and fructose-6-phosphate is diverted into a signaling pathway [234,248]. An enzyme called glutamine/fructose 6-phosphate amidotransferase converts fructose 6-phosphate to glucosamine 6-phosphte and finally to UDP N-acetylglucosamine (Figure 9). UDP-N-acetylglucosamine is used as a substrate for serine and tyrosine glycosylation of several transcription factors, such as plasminogen activator inhibitor-1 (PAI-1) and transforming growth factor-β1 (TGF-β1), leading to microvascular complications in diabetes [263,264].

All four of these mechanisms are triggered by the overproduction of superoxide by the mitochondrial electron transport chain [265,266,267]. Glucose uptake is assisted by insulin-independent GLUTs, GLUT1 and GLUT3, the latter being abundant in neurons [268,269,270]. After glucose enters the neurons, glycolysis and the tricarboxylic cycle (TCA) are saturated, funneling electron donors into the electron transport chain. Also, the lactate derived from glycolysis in astrocytes can enter the neurons to be converted to pyruvate, to power the TCA cycle. The electron transport chain is overloaded and, as a result, the voltage gradient across the mitochondrial membrane increases until the electron transfer inside complex III is blocked. As a result, electrons from coenzyme Q are donated one at a time to molecular oxygen, producing superoxide [251,271]. Experiments conducted by Michael Brownlee [249] showed that the mitochondrial electron transport chain is the source of hyperglycemia-induced superoxide, which is the initial ROS formed. In the absence of the mitochondrial electron transport chain (in mitochondrial DNA-depleted endothelial cells), the polyol pathway, AGE formation, PKC or the hexosamine pathway are not activated by hyperglycemia [249]. Also, overexpression of uncoupling protein 1 (UCP-1) or MnSOD showed that hyperglycemia did not activate any of these pathways [256]. Considering all the observations, Brownlee proposed a single key trigger event—the inhibition of GAPDH by superoxide overproduction due to hyperglycemia [249]. Because of GAPDH’s inhibition, all the glycolytic intermediates that are upstream of GAPDH increase: glyceraldehyde-3-P activates the AGE pathway and the PKC pathway, fructose-6-P increases the flux through the hexosamine pathway and glucose increases the flux into the polyol pathway.

GADPH inhibition is the result of its polyADP-ribosylation by poly (ADP-ribose) polymerase PARP, which was activated due to DNA strand breaks caused by excessive mitochondrial superoxide production [272]. PARP is a DNA repair enzyme, residing exclusively in the nucleus in an inactive form. ROS generated in the mitochondria induce DNA strand beaks, activating PARP. The activated PARP splits NAD^+^ molecule into nicotinic acid and ADP-ribose and makes polymers of ADP-ribose and attaches them to several nuclear enzymes. It seems that GAPDH has a critical role in DNA repair and goes in and out of the nucleus all the time [273,274]. NAD^+^ depletion slows the rate of glycolysis, electron transport and ATP formation.

Mitochondria ROS overproduction is also the result of increased fatty acid flux from the adipocytes into endothelial cells, because of insulin resistance. These fatty acids are then oxidized in the mitochondria, producing ROS by the same mechanisms as hyperglycemia [266].

Impairment of mitochondrial function reduces the ATP synthesis and affects insulin secretion. Normally, the rise in the ATP/ADP ratio leads to the closure of ATP-sensitive K^+^ channels, which results in the depolarization of voltage-sensitive Ca^2+^ channels and subsequently triggers the exocytosis of insulin-containing secretory vesicles [275]. Specific inhibitors targeting the mitochondrial respiratory chain complexes were found to suppress insulin release from pancreatic islet cells [250].

Superoxide also activates UCP-2-mediated proton leaks, leading to changes in the level of an important transcription factor for the insulin promoter, pancreas duodenum homebox-1 (PDX-1), necessary for insulin gene expression [276]. Activation of UCP-2 also decreases the ATP/ADP ratio, contributing to a reduced insulin secretory response [277]. It is possible that hyperlipemia (in obese patients) causes also a superoxide-mediated activation of UCP-2 and thus a loss in insulin secretion (UCP-2 knockout mice maintain insulin secretion after long-term incubation with palmitic acid).

With aging, increased ROS production, mitochondrial DNA damage accumulation and progressive respiratory chain dysfunction affect the mitochondrial function and determine the insulin resistance seen in T2D [250].

## 3. Conclusions

PD and T2D, despite their distinct clinical presentations, share several underlying mechanisms, including chronic inflammation, mitochondrial dysfunction, impaired protein handling and ferroptosis. The excessive accumulation of ROS and the iron-dependent lipid peroxidation, associated with a lack of antioxidative protection, especially due to aging, represents a crucial point of convergence in the pathology of both diseases. Targeting iron metabolism and ferroptosis could provide dual benefits in treating both diseases. Iron chelators and antioxidants that inhibit ferroptosis hold promise as potential therapeutic agents. Moreover, identifying biomarkers of ferroptosis could aid in the early diagnosis and monitoring of therapeutic efficacy. Additionally, repurposing existing drugs that modulate these pathways might offer effective strategies for managing both PD and T2D and improve the quality of life for millions of affected individuals worldwide.

## Figures and Tables

**Table 1:** Different types of regulated cell death and their morphological features.

Type	Morphological Features
Ferroptosis	Cell membrane: lack of rupture and blebbing of the plasma membrane; rounding-up of the cell Cytoplasm: small mitochondria with condensed mitochondrial membrane densities, reduction or vanishing of mitochondria crista, as well as outer mitochondrial membrane rupture Nucleus: normal nuclear size and lack of chromatin condensation
Apoptosis = regulated cell death triggered by disturbances in the extracellular or intracellular environment	Cell membrane: plasma membrane blebbing; rounding-up of the cell Cytoplasm: retraction of pseudopods; reduction in cellular volume Nucleus: reduction in nuclear volume; nuclear fragmentation; chromatin condensation
Necroptosis = regulated inflammatory cell death, a form of necrosis that activates innate immune responses	Cell membrane: lack of change Cytoplasm: accumulation of double-membraned autophagic vacuoles Nucleus: lack of chromatin condensation
Autophagy = natural and regulated biological process that disassembles unneeded or malfunctioning cell components	Cell membrane: lack of change Cytoplasm: accumulation of double-membraned autophagic vacuoles Nucleus: lack of chromatin condensation

## Data Availability

Not applicable.
